# The Needs of Older Adults With Disabilities With Regard to Adaptation to Aging and Home Care: Questionnaire Study

**DOI:** 10.2196/16012

**Published:** 2020-10-26

**Authors:** Laiyou Li, Ning Sun, Libo Yu, Xiaoxin Dong, Jing Zhao, Yuchen Ying

**Affiliations:** 1 Ningbo College of Health Sciences Ningbo China

**Keywords:** community, disability, older people, adaptation to aging, influence factor

## Abstract

**Background:**

The home environment is an important means of support in home-based care services for older people. A home environment that facilitates healthy aging can help older adults maximize their self-care abilities and integrate and utilize care resources. However, some home environments fail to meet the needs of older adults with disabilities.

**Objective:**

This paper aimed to study the needs of older adults with disabilities with respect to adaptation to aging, and to analyze the associations of individual factors and dysfunction with those needs.

**Methods:**

A questionnaire survey was administered to 400 older adults with disabilities from 10 communities in Ningbo City, Zhejiang Province, China. The survey was conducted from August 2018 to February 2019.

**Results:**

A total of 370 participants completed the survey. The proportion of participants with mild dysfunction was the highest (128/370, 34.59%), followed by those with extremely mild (107/370, 28.92%), moderate (72/370, 19.46%), and severe (63/370, 17.03%) dysfunction. The care needs of older adults with extremely mild and mild dysfunction pertained primarily to resting, a supportive environment, and transformation of indoor activity spaces. The care needs of older adults with moderate dysfunction pertained mainly to resting and renovation of bathing and toilet spaces. Factors influencing the needs of older adults with disabilities were dysfunction (*P*=.007), age (*P*=.006), monthly income (*P*=.005), and living conditions (*P*=.04).

**Conclusions:**

The needs of older adults with disabilities varied by the degree of dysfunction, and many factors influenced these needs in the community. These findings may provide a scientific basis for developing community-specific aging-related adaptation services for older adults with disabilities in the future.

## Introduction

### Background

In 2014, it was estimated that there were 40 million older people with disabilities in China; this figure has been predicted to reach 61.68 million by the end of 2030. In 2016, at least 60-70 million people in China were reported to need long-term care [1]. Home care refers to older adults living and receiving care at home rather than at an assisted living facility, which is currently the most common pension model in China [2]. A total of 10 governmental departments have published policy documents to promote the development of home care services [3]. The home environment is an important means of support for home-based care services. A home environment that facilitates healthy aging can help older adults maximize their self-care abilities and integrate and utilize care resources. A key aspect of home care services is the assessment of aging needs in the home environment. Some home environments fail to meet the growing needs of older adults, especially of those with disabilities. Many home environments have design problems, such as the lack of barrier-free transportation systems, bathroom space security, and auxiliary facilities; unreasonable design and spatial layout; and imperfect alarms and help sign systems [4]. Thus, the needs of older adults with disabilities during adaptation to aging in their home environment must be explored urgently.

Chinese researchers have conducted a large number of theoretical and practical studies of design transformation, focusing on the living environments of older adults and the need for redesign of their residences, and have achieved relatively significant results. Saiquan [5] suggested that through adaptation to aging, one-third to half of casualties among older adults can be prevented, and older adults can remain in their original housing for more than 10 years with adequate self-care. Xiaoyun [6] examined the environmental planning strategies for older adult–friendly communities, including the suitability of residential units, open spaces, transportation; service facilities for older people; and renovations for old buildings. Yanwei and Jiayan [7] assessed the suitability of existing residential areas with respect to adaptation to aging by considering different perspectives, such as those of developers, designers, and buyers. Outdoor activity needs of older adults were reported, and the study proposed suitable design principles for the outdoor environment, including different types of activity spaces, outdoor facilities, and garden elements [7]. Shenmao and Jijun [8] conducted a study from the perspective of the residential fitness design and discussed the “dwelling residential design” in the United States, the “House Design Guide for Longevity Society” in Japan, and “Residential Design for the Elderly” in China [8]. Most theoretical and practical reviews on the construction, renovation, and design of home environments suited to the needs of older adults, both in China and worldwide, are aimed at solving these problems in urban contexts.

Aging of the population is an inevitable result of social development. Most developed countries became societies with larger proportions of older people in the 1960s and 1970s. Early transformation into such a society has led to early emergence of theoretical studies and practical exploration of housing designs suited to the needs of the older population in these countries [9-11]. Many studies suggest that aging-appropriate transformations of home environments for older adults can help prevent falls, delay functional decline, improve quality of life, and save costs incurred in hiring care labor [12-15]. According to studies conducted across various countries, the degree of such transformation is determined by both objective and subjective factors. Objective factors refer to the material conditions of the environment. Subjective factors include individual behavior, life experience, and awareness of the living environment. Developed countries attach greater importance to the problem of suitable accommodation for older people in the home environment. The costs of aging-appropriate renovations are also covered through state subsidies or insurance in Sweden, Germany, Japan, and other developed countries [16-19].

### Objectives

The aim of this study was to determine the needs of older adults with disabilities with respect to adaptation to aging in 10 communities in Ningbo City and to analyze the association of individual factors and levels of dysfunction with those needs.

## Methods

### Study Design

A correlational, cross-sectional design was adopted, and questionnaires were used for data collection.

#### Participant Selection

A total of 400 older adults with disabilities were identified and selected from August 2018 to February 2019 by convenience sampling from 10 communities in 5 districts of Ningbo: Jiangbei, Yinzhou, Haishu, Beilun, and Zhenhai. Samples from each district comprised 80 older adults with disabilities. A total of 400 questionnaires were administered to the participants, and 370 valid questionnaires were recovered.

The selection criteria were as follows: age ≥60 years, permanent residence in Ningbo, living in the community for more than 6 months, diagnosed with a disability based on the Daily Living Activity Scale, and volunteering to participate in the study. The exclusion criteria were as follows: severe cognitive dysfunction, unconsciousness, and difficulties in understanding instructions.

#### Sample Size Calculation

The study mainly examined the correlation between individual factors and the need for adaptation to aging. Multifactor analysis methods were applied. Based on relevant research, it was estimated that 25 variables could be entered into the model. The required sample size was estimated to be at least 10-15 times the number of variables entered into the model, thus requiring 375 research participants. The follow-up loss rate was calculated at 1%. Therefore, the required sample size was estimated at 400 people.

#### Data Collection

Two investigators underwent unified training. Data were collected by in-person interviews. All participants stayed at home or in nursing homes and were included in this study after a scheduled meeting arranged by the nursing home and community service center managers. The investigators explained the research objectives and methods to individuals who met the inclusion criteria and obtained consent and cooperation from participants and their families. Consenting participants received an envelope containing a packet with the questionnaires. Participants completed the questionnaires immediately upon receipt and placed them in the envelope for collection by the investigators. To ensure anonymity, each completed questionnaire was assigned a code number.

### Study Measures

The following 3 questionnaires were used in this study: the Demographic Data Questionnaire, the Activity of Daily Living Scale, and the Questionnaire on Needs for Adaptation to Aging.

#### The Demographic Data Questionnaire

This questionnaire collected information on participants’ age, gender, education level, monthly income, marital status, residence status, and payment method of medical expenses.

#### The Activity of Daily Living Scale

This scale was developed by American researchers in 1969 to determine the degree of dysfunction among older adults with disabilities. It consists of 14 items that are rated on a 4-point rating scale, with responses ranging from “can do it yourself” to “cannot do it yourself” [20,21]. The scores range from 14 to 56, with higher scores indicating higher levels of dysfunction. Scores ≤20 indicate completely normal function. Scores >20 indicate varying degrees of dysfunction (21-30: very mild; 31-40: mild; 41-50: moderate; >50: severe). Cronbach alpha for this scale is .92, and the content validity index is 0.86.

#### Questionnaire on Needs for Adaptation to Aging

This questionnaire comprises 9 first-level indicators and 55 secondary indicators. The first-level indicators include entrances and exits (12 items), indoor activities (6 items), toilet (3 items), bathing (5 items), modification/renovation (3 items), rest (5 items), preparation/meal (6 items), laundry (4 items), and supportive environment (11 items). Each item is rated on a 5-point Likert scale, with scores ranging from 1 to 5 points. The higher the score, the higher the need for adaptation to aging. The Cronbach alpha of the questionnaire is .96, and its content validity index is 0.95.

### Data Analysis

SPSS 21.0 (IBM Corp) was used for data analysis after the logistic test. A *P* value <.05 was considered to be statistically significant. Mean (SD), frequencies, and percentages were used to describe demographic data, dysfunction, and the needs of older adults with disabilities for adaptation to aging. The association of individual factors and dysfunction with the needs of the participants was analyzed by multiple linear regression analysis.

### Ethical Considerations

This study was approved by Ningbo College of Health Sciences (NBWY-030). All participants were included in this study following the meeting schedules provided by nursing home and community service center managers, and they were briefly informed of their right to withdraw at any time. Older individuals with disabilities who consented to or were authorized to participate in the study received the questionnaire.

## Results

A total of 400 questionnaires were distributed, and 378 were completed (recovery rate of 94.5%). There were 370 valid questionnaires (effective recovery rate of 92.5%).

Of the participants, females constituted the majority (252/370, 68.1%). Participants’ ages ranged from 60 to 91 years, and the average age was 70.21 (SD 5.98) years. The majority of the participants (253/370, 68.4%) were married, and 29.7% (110/370) were widowed. Regarding education levels, the majority of the participants had completed junior secondary school (141/370, 38.1%), while 28.6% (106/370) had completed high school. Most of the older adults with disabilities (281/370, 75.9%) lived with their families. Most participants’ (261/370, 70.5%) monthly income ranged from 1001 yuan to 3000 yuan. Majority of the participants (254/370, 68.6%) were mainly dependent on urban medical insurance. Table 1 shows the degrees of dysfunction among the study participants.

**Table 1:** Degree of dysfunction among the study participants (N=370).

Degree of dysfunction	Activity of Daily Living Scale scores, mean (SD)	Values, n (%)
Extremely mild	24.54 (1.90)	107 (28.92)
Mild	32.69 (2.11)	128 (34.59)
Moderate	42.26 (1.66)	72 (19.46)
Severe	51.48 (1.71)	63 (17.03)

In cases of participants with extremely mild and mild dysfunction, needs for adaptation to aging primarily pertained to resting, a supportive environment, and suitable spaces for indoor activities. Needs of those with moderate and severe dysfunction mainly pertained to resting and suitable bathing and toilet spaces (Table 2).

**Table 2:** Adaptation needs of the participants by degree of dysfunction (N=370).

Needs for adaptation to aging as per the questionnaire	Degrees of dysfunction			
	Extremely mild	Mild	Moderate	Severe
Entrances and exits, score points, mean (SD)	4.29 (0.71)	4.31(0.72)	4.33 (0.73)	4.35 (0.74)
Indoor activities, score points, mean (SD)	4.44 (0.76)	4.45 (0.75)	4.45 (0.76)	4.47 (0.75)
Toilet space, score points, mean (SD)	4.41 (0.79)	4.43 (0.78)	4.49 (0.79)	4.51 (0.78)
Bathing space, score points, mean (SD)	4.42 (0.80)	4.42 (0.81)	4.50 (0.83)	4.52 (0.82)
Modifying/renovation, score points, mean (SD)	4.41 (0.88)	4.43 (0.87)	4.45 (0.89)	4.47 (0.87)
Resting, score points, mean (SD)	4.48 (0.81)	4.50 (0.82)	4.53 (0.84)	4.55 (0.83)
Meals/meal preparation, score points, mean (SD)	4.40 (0.86)	4.42 (0.85)	4.45 (0.87)	4.47 (0.88)
Laundry, score points, mean (SD)	4.35 (0.92)	4.37 (0.91)	4.39 (0.93)	4.40 (0.91)
Supportive environment, score points, mean (SD)	4.47 (0.78)	4.49 (0.77)	4.48 (0.79)	4.50 (0.80)

The correlation coefficients between participants’ demographic characteristics and adaptation to aging are shown in Table 3. Age, education level, monthly income, residence status, and the degree of dysfunction are significantly correlated with adaptation to aging (Table 3).

**Table 3:** Correlation between participants’ demographic characteristics and adaptation to aging.

Variable	Correlation coefficient (*r*) for adaptation to aging	*P* value
Age	0.335	.02
Gender	0.217	.08
Education level	0.296	.04
Monthly income	0.369	.03
Marital status	0.215	.12
Residence status	0.316	.04
Payment method	0.235	.09
Degree of dysfunction	0.398	.008

With significantly correlated demographic characteristics and dysfunction as the independent variables, factors affecting needs for adaptation to aging were analyzed by stepwise regression. By the degree of influence, dysfunction ranked the highest, followed by age, monthly income, and living conditions (Table 4).

**Table 4:** Factors affecting needs of the participants for adaptation to aging.

Variable	Sample regression coefficient (*b*)	Beta level (β)	*t* test scores	*P* value
Age	4.68	.22	4.98	.006
Residence status	–2.96	–.06	–2.45	.04
Monthly income	3.98	.16	–5.38	.005
Degree of dysfunction	9.86	.49	8.85	.007

## Discussion

This study conducted a comprehensive questionnaire survey of the needs of older adults with disabilities with respect to adaptation to aging in 10 communities in Ningbo City. The results showed that the proportion of older adults with disabilities and mild dysfunction was the largest. The needs of older adults with disabilities varied with different degrees of dysfunction, and many factors influenced their needs in the community at large. The results of this study provide a scientific basis for the provision of targeted rehabilitation services to older people with disabilities in order to improve their quality of life.

### The Status of Dysfunction Among Older Adults With Disabilities in Ningbo

In total, 34.59% (128/370) of older adults with disabilities reported mild dysfunction (Table 1). The proportion of older adults with disabilities in this study is higher than that in a study conducted in Shanghai [22]. The main reason for this difference could be that Shanghai’s economic development and levels of medical care are more advanced than those of Ningbo. Thus, older adults with disabilities and their families in Shanghai have more opportunities to choose from good-quality health care resources in case of major diseases, and this access significantly reduces the extent of dysfunction experienced by them. Furthermore, healthy behavior and the degree of health awareness among older adults depend on their level of cultural literacy. Older people with high cultural literacy are more likely to pay attention to their health and seek timely medical treatment, thus significantly reducing the incidence of dysfunction. Moreover, Shanghai’s regional medical security and long-term care insurance systems are more effective than those of Ningbo. Further improvements of both systems can improve the quality of life among older adults with disabilities in Ningbo.

### The Needs of Older Adults With Disabilities With Regard to Adaptation to Aging

The needs of older adults with disabilities and extremely mild or mild dysfunction mainly pertained to resting, a supportive environment, and suitable indoor activity spaces. In most cases, older people with extremely mild or mild dysfunction can eat, dress, and wash. They can take care of themselves in their daily lives and are less dependent on others. Thus, there is a high demand for a supportive environment and broader, safer spaces suitable for their indoor activities. Participants reported that they wish to switch on the lights around their beds, as indoor lighting can help them see their indoor environment clearly. They also expressed the need for sufficiently audible phone and doorbell sounds. These findings are consistent with those of another study [23].

The needs of older adults with disabilities and moderate or severe dysfunction mainly pertained to resting and safe bathing and toilet spaces. Most older people with moderate or severe dysfunction lose their daily living ability and need to rely on caregivers. Studies have shown that older people with moderate or severe dysfunction usually have 2 or more different diseases, which may restrict their functional activities and warrant complete bed rest. Therefore, they hope to have a safe bed rest environment, which includes installation of handrails on the bed to help them get up. Moreover, they hope to have a safe bathing and toilet environment, which includes handrails and alarms in the toilet and bathroom to get help when they lose their balance or fall. This finding is also consistent with the findings of another study [24].

### Factors Affecting the Needs of Older Adults With Disabilities With Regard to Adaptation to Aging

The results of the present study showed that the factors influencing adaptation to aging among older adults with disabilities were degree of dysfunction, age, monthly income, and living conditions. The standardized regression coefficient of dysfunction degree was 0.49, which had the highest impact on the need for adaptation to aging. This indicates that the older the person experiencing dysfunction, the greater their need for adaptation to aging. Older adults with disabilities having greater dysfunction and poor quality of life hope to enhance the quality of their lives by improving their living environment.

The results of this study also suggest that the older the person, the higher the benefits they receive from adaptation to aging. As a person grows older, the functions of various organs in their bodies decline, resulting in 2 or more diseases and other complications. Long-term physical illness leads to psychological problems and reduced ability to engage in social activities. A study showed that the need for such adaptation increases with aging [25].

Furthermore, the standardized regression coefficient for monthly income was 0.16, indicating that the lower the monthly income, the lower the demand for adaptation to aging. As older adults with disabilities may have multiple diseases, they need long-term medication or rehabilitation, resulting in an increasing economic burden. Families experiencing financial strain are less likely to take necessary actions for adaptation to aging and to improve the residential environment of older adults with disabilities. In case of older adults with low monthly incomes, without any financial support from other sources, the income is mainly spent on dietary needs and medical treatment, rather than meeting needs specific to adaptation to aging. Other studies have shown [25] that the higher the monthly income, the stronger the willingness to adapt. Therefore, the state needs to consider the low-income groups among the older population with disabilities when formulating policies for home-based care services.

The standardized regression coefficient for living conditions was –0.06, that is, the needs of older persons living alone are high. On the contrary, older people with disabilities have fewer needs if they have family members to take care of them. The aforementioned study [25] reported that older people who do not live with their children have a strong willingness to transform their living environments, as they do not have caregivers. Moreover, there are more risk factors in such living environments. Thus, by transforming their environment, they can improve their safety. With the development of society, there has been an increase in the prevalence of “4-2-1” and “4-2-2” family structures, which indicates an increase in the number of older people who live alone and experience the empty nest syndrome. Challenges in providing long-term care to such older adults are more prominent. Therefore, the results of this study suggest that the governments or the relevant agencies should strengthen the policy guidance for these special groups when formulating policies to help older population living alone to adapt to aging and improve their quality of life.

There are some limitations to this study. First, a convenience sample was used. Second, data were obtained only from the South of China, limiting the generalizability of the findings. Therefore, in the future, the relationships between adaptation needs of older people with disabilities and the factors influencing these needs should be examined by conducting a cohort study on a more diverse sample.
